# The COUNTDOWN Study Protocol for Expansion of Mass Drug Administration Strategies against Schistosomiasis and Soil-Transmitted Helminthiasis in Ghana

**DOI:** 10.3390/tropicalmed3010010

**Published:** 2018-01-22

**Authors:** Suzy J. Campbell, Mike Y. Osei-Atweneboana, Russell Stothard, Artemis Koukounari, Lucas Cunningham, Samuel K. Armoo, Nana-Kwadwo Biritwum, Margaret Gyapong, Eleanor MacPherson, Sally Theobald, Maame Esi Woode, Jahangir Khan, Louis Niessen, Emily R. Adams

**Affiliations:** 1 Department of Parasitology, Liverpool School of Tropical Medicine, Pembroke Place, Liverpool L3 5QA, UK; suzy.campbell@evidenceaction.org (S.J.C.); russell.stothard@lstmed.ac.uk (R.S.); lucas.cunningham@lstmed.ac.uk (L.C.); 2 Department of Environmental Biology and Health, Council for Scientific and Industrial Research—Water Research Institute, P.O. Box M 32, Accra 102001, Ghana; oseiatweneboana@yahoo.co.uk (M.Y.O.-A.); samuel.k.armoo@gmail.com (S.K.A.); 3 Department of Clinical Sciences, Liverpool School of Tropical Medicine, Pembroke Place, Liverpool L3 5QA, UK; Artemis.Koukounari@lstmed.ac.uk (A.K.); maame.woode@lstmed.ac.uk (M.E.W.); jahangir.khan@lstmed.ac.uk (J.K.); louis.niessen@lstmed.ac.uk (L.N.); 4 Neglected Tropical Diseases Control Programme, Ghana Health Service, Accra 102001, Ghana; nkadbiritwum@gmail.com; 5 Dodowa Health Research Centre, Ghana Health Service, Dodowa 116001, Ghana; mgyapong@uhas.edu.gh; 6 Department of International Public Health, Liverpool School of Tropical Medicine, Pembroke Place, Liverpool L3 5QA, UK; eleanor.macpherson@lstmed.ac.uk (E.M.); sally.theobald@lstmed.ac.uk (S.T.)

**Keywords:** schistosomiasis, haematobium, mansoni, soil-transmitted helminthiasis, access, chemotherapy, praziquantel, albendazole, Ghana

## Abstract

(1) *Background:* Current international policy for schistosomiasis and soil-transmitted helminthiasis (STH) control emphasises mass administration of deworming drugs in school-based programmes. However, this approach is insufficient to control the transmission of these diseases, and their burden in non-school cohorts is recognised, albeit under-researched. This research will investigate the feasibility and acceptability of expanding access to praziquantel (PZQ) against schistosomiasis, and albendazole (ALB) against STH, to communities in selected transmission settings in Ghana. (2) *Methods:* A three-site longitudinal study will be implemented to investigate the effectiveness of expanding treatment strategies for PZQ and ALB to community members. In the context of community mass drug administration (to preschool children, school non-attending children, and adults, including pregnant women), the intervention will be assessed in a random sample of community members, at baseline with follow-up at 6, 12, and 18 months. In each community, 658 participants will be enrolled, and 314 followed up at each time point. The primary outcome measure is the prevalence of infection of *Schistosoma haematobium* and/or *S. mansoni* at study endpoint, as assessed by longitudinal surveys. Secondary outcomes are to quantify the infection of schistosomiasis and STH infections in non-treated cohorts, reductions in prevalence of STH, and intensity of schistosomiasis and STH, and treatment coverage. Nested within this study will be qualitative, cost-benefit, and cost-effectiveness evaluations that will explore accessibility, feasibility, and economic impact of expanded treatment from different complementary perspectives. (3) *Discussion:* Using a multidisciplinary approach, this study will generate evidence for improved availability, acceptability, affordability, and accessibility to deworming drugs against schistosomiasis and STH to individuals and communities in Ghana. This is likely to have considerable research, programmatic, and political value to contribute evidence for national programme policy development within Ghana, and, more broadly, World Health Organization policy development.

## 1. Background

Schistosomiasis (*Schistosoma mansoni* and *S. haematobium*) and soil-transmitted helminthiasis (STH; *Ascaris lumbricoides*, *Trichuris trichiura*, *Necator americanus*, *Ancylostoma duodenale*, and the less-researched *Strongyloides stercoralis*) infections constitute a major public health problem and share the majority burden of disease from the group of neglected tropical diseases (NTDs) [1]. Globally, schistosomiasis and STH cause an estimated disease burden exceeding eight million disability-adjusted life years [2]. Infections contribute heavily to intestinal damage, anaemia, impaired physical, and mental growth in children, and, for schistosomiasis, urinary and genital organ damage with important differences in males and females [3,4]. In schistosomiasis-endemic areas, symptoms of both female genital schistosomiasis (FGS) and male genital schistosomiasis (MGS) can manifest after many years of infection, commencing from preschool-aged exposure [5]. Unfortunately, assessment of pathological changes and symptoms in all of the cohorts is under-researched and these disorders are under-diagnosed in the developing world [6]. FGS leads to stigma and social isolation, with symptoms including menstrual irregularity, menorrhagia (heavy bleeding), pain during or after sexual intercourse (dyspareunia), post-coital bleeding, ectopic pregnancy, miscarriage, and sub-fertility [7,8]. Schistosomiasis and STH are endemic in many countries, including Ghana, with STH being prevalent in most regions, and schistosomiasis proximal to water bodies containing *Bulinus* or *Biomphalaria* snail intermediate hosts [9]. People contract schistosomiasis from contact with water containing *Schistosoma* cercariae. STH life cycles involve environmental contamination with faeces containing helminth eggs; therefore, major risk factors include poor hygiene, sanitation, and access to clean water [10,11].

Mass drug administration (MDA) programs using praziquantel (PZQ) against schistosomiasis, and benzimidazole drugs (albendazole (ALB) or mebendazole (MBD), according to country) against STH, primarily focus on periodic treatment of school-aged children in school-based programmes (with some provision of STH treatments to preschool-aged children in some areas) [12]. Historically, this has been based on the assumptions that most attributable morbidity would be reduced by treating the school-aged cohort; that potential anthelmintic resistance is held in check by focusing on the school-aged cohort; and, that utilisation of existing school infrastructure is the most feasible and cost-effective means of delivering preventive chemotherapy programmes in resource-constrained settings [13,14]. However, morbidity in non-school cohorts does occur, meaning that deworming school-aged children alone is an imperfect strategy, particularly after years of MDA programmes [5,13]. With the current strategy, the burden of disease in preschool-aged and adult cohorts is therefore inadequately researched, and subsequently, under-prioritised in health programmatic planning. Inequity of access to deworming drugs by children who are not in school (for a variety of reasons, including the interplay of poverty and gender norms), and adult cohorts is a recognised problem [15,16,17,18,19,20]. Data demonstrating prevalence in vulnerable groups, treatment benefits to these groups, and feasibility of expanding access, are required to effect policy change. Despite considerable progress, at present WHO 2020 targets of treating 75% of school-aged children annually for morbidity control [21] are not yet being achieved [22], and, consequentially, millions of individuals suffer long-term debility, which could and should be avoided. In 2015, reported deworming coverage of school-aged children was 42.2% for schistosomiasis, 63.3% for STH; and, coverage of preschool-aged children for STH was 48.2% [23].

Assessing the effectiveness of increasing MDA access to broader community groups is a considerable global research priority. Thus far, research has focused on mathematical modelling studies [24,25,26,27] for STH, which indicate benefits of expanded treatment strategies. Impetus to provide an epidemiological evidence base is growing. A large-scale randomised controlled trial (RCT) investigating alternative treatment strategies for STH is under way, with anticipated results by 2018 [28]. An additional multi-site series of cluster RCTs will commence in 2017, to determine the feasibility of interrupting STH transmission and develop an implementation plan for scale-up in Malawi, Benin, and India [29]. Both of the initiatives focus on treatment strategies for STH. For schistosomiasis, the Schistosomiasis Consortium for Operational Research and Evaluation (SCORE) is undertaking studies in Kenya, Mozambique, Tanzania, Cote d’Ivoire, and Nigeria to investigate gaining and sustaining control in moderate-heavy transmission settings [30]; whilst extremely beneficial, these studies are assessing outcomes in school-aged children and do not longitudinally follow up the same individuals. Also associated with SCORE, studies in Zanzibar [31,32] have focussed on expanded treatment strategies to understand the thresholds and combined activities that are needed for elimination. This agenda-defining work is, however, focussed in a country that has experienced three decades of dedicated schistosomiasis control activities. Therefore, multidisciplinary work in other endemic countries is needed. Widespread elimination of schistosomiasis will almost certainly need the integrated use of preventive chemotherapy, snail control, behavioural modification, water, sanitation and hygiene (WASH) improvements, and perhaps eventually a prophylactic or transmission-blocking vaccine [9]. A recent meeting, ‘NTDs: Women and girls in focus’, discussed the importance of better understanding ways to ensure MDA programmes reach and reflect the needs of all groups by gender and age [33]. 

In Ghana, annual MDA is well-established and political motivation high, making it an excellent site in which to research concentrated treatment strategies. Implementation research will explore the feasibility and acceptability of expanding treatment to preschool-age children, out-of-school (school-aged) children, and adults, including pregnant women in selected community sites. The aim is to demonstrate that PZQ and ALB treatments are needed and can be up-scaled and delivered to these demographical groups (which, particularly for PZQ, are not presently targeted within national control programmes), achieving adequate treatment coverage, and resulting in more rapid declines in infection and disease state than annual school-based treatment. Research components include epidemiological, parasitological, social science, and health economics analyses, conducted across an international, multi-stakeholder environment. This study is anticipated to reduce prevalence and intensity of infection of schistosomiasis and STH, increase treatment coverage, and provide evidence to ensure more equitable access to vulnerable groups. In turn, this will help to strengthen the broader health system and generate evidence for improved availability, acceptability, affordability, and accessibility of these NTD interventions to individuals and communities. 

## 2. Design/Methods

A multi-site longitudinal study will be conducted in Ghana to investigate the effectiveness of expanding treatment strategies for PZQ and ALB to community members in a peri-urban setting, where the risk of schistosomiasis and STH is considerable. The intervention will be assessed at 6, 12, and 18 months. Nested within this study will be qualitative and socio-economic evaluations that will explore the accessibility, feasibility, and economic impact of expanded treatment from different complementary perspectives. 

The specific objectives are:

1. To conduct targeted baseline parasitological and epidemiological surveys to describe infection levels of schistosomiasis and STH, and associated WASH and morbidity indicators;
2. To assess expanded annual community treatment in reducing prevalence of schistosomiasis and STH;
3. To assess community understanding of the current MDA programme and its strengths and weaknesses and economic aspects of whole community treatment;
4. To assess the acceptability and feasibility of introducing an alternative treatment strategy to the current treatment regimen from different perspectives;
5. To investigate different levels of access and factors influencing scale-up of PZQ and ALB treatment in areas with limited treatment coverage.

## 3. Study Area and Site Selection

**Figure 1:**
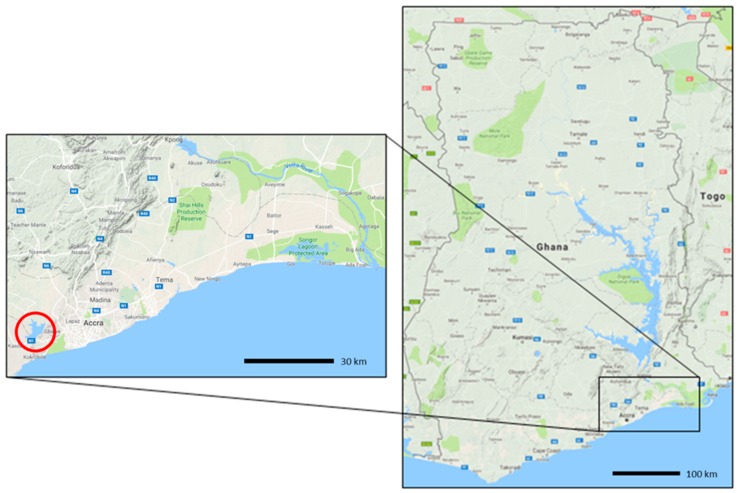
Map of Ghana showing location of study regions; images adapted from Google maps.

Due to the highly spatially focal nature of schistosomiasis, regions have been purposively selected according to known endemicity (based on National NTD Control Programme data). Communities within these regions have been selected based on prevalence of at least 10% of schistosomiasis infection (being *S. mansoni* or *S. haematobium*, or both), and proximity to water bodies containing intermediate snail hosts that has previously been ascertained (JRS unpublished). Typically, STH are co-endemic with schistosomiasis in these regions, but are not the basis for site selection, which is the focused on a peri-urban setting surrounding a manmade reservoir. In Ghana, most health care is provided by the government and is largely administered by the Ministry of Health and Ghana Health Service (GHS). There are five levels of health care provision: health posts, health centres and clinics, district hospitals, regional hospitals, and tertiary hospitals. Administratively, GHS is organized at national, regional, and district levels [33]. There are 110 health districts. For Ghana, three communities were selected in Ga South District (Greater Accra Region) (Figure 1). Due to the highly focal nature of disease, we will use routine data collected by GHS and CSIR to serve as controls.

## 4. Study Design

This longitudinal study will comprise baseline and follow-up surveying of the same individuals. Baseline surveying will involve screening urine and stool samples from a random selection of participants to assess infection prevalence and intensity, and conducting questionnaires to determine associations with WASH and morbidity from both schistosomiasis and STH. Qualitative focus group discussions and in-depth interviews will be held with community members and key informants to assess the understanding of the current MDA strategy, its strengths, and weaknesses, and to identify the feasibility and acceptability of expanding treatment in communities. Comparisons will be made: (i) after 6, 12, and 18 months; and, (ii) to the provision of annual MDA in the regular National School-Based Deworming Programme in schools in the same health districts, at the same time points as a means of comparison to the existing school-based deworming programme in similar prevalence settings. Drug treatment coverage for each survey will also be measured.

**Figure 2:**
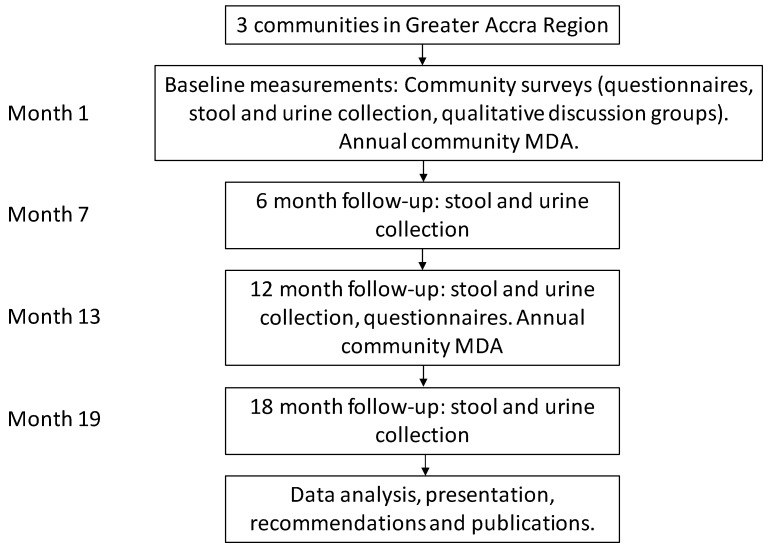
Diagram showing the study design of the expanded treatment intervention survey.

This study will involve expanding access to PZQ+ALB treatment for the broader population, including preschool-aged children, out-of-school school-aged children, and adults, including pregnant women. An annual community-based PZQ+ALB treatment will be provided by community health nurses and trained field staff, with cross-checking of enrolled school-aged children so as not to double-up treatments that have been provided in school-based MDA. Treatment will be provided at baseline and 12 months, and the associated reductions in prevalence and intensity of schistosomiasis and STH will be assessed (Figure 2). Parasitological examinations and questionnaires will be conducted on a random sample of participants to determine associations with morbidity from both schistosomiasis and STH, and investigate the feasibility of scale-up. An experienced and trained social science team will conduct qualitative interviews with teachers that are involved in administering annual MDA, and focus group discussions with purposively selected groups of participants by gender, age, and socio-economic status, as well as parents and caregivers.

For comparison purposes, three school-based sites in the same health districts as the study sites will be selected. In these schools, a random sample of school-aged children who currently receive annual MDA will be selected to participate in parasitological examinations and questionnaires, to enable comparison to the national school-based deworming programme. These data and treatment coverage data, available from the national NTD Control Programme, will be analysed for schools in the same health districts that are not included in the surveys. Due to ethical concerns about conducting parasitological testing for schistosomiasis in communities without the provision of treatment, and where there is no existing opportunity for treatment referral, community sites will not be used for comparison purposes in these studies. This is supported by school sites being the existing MDA unit.

## 5. Intervention

All community members with valid consent will be offered PZQ+ALB, delivered by community health nurses/trained field staff. This will involve community sensitisation within the selected villages, and then enrolment of participants with exclusion of school-enrolled children who received MDA, children less than one year of age, and (for ALB) women in their first trimester of pregnancy in accordance with current WHO recommendations. Participants in the community-based survey will be invited to join a whole community treatment MDA day, where they will receive a baseline and a 12-month treatment of 600 mg PZQ at 40 mg/kg body weight and a single dose of 400 mg ALB. The research team will conduct the parasitological examinations and questionnaires, and the whole community MDA will take place after all of the samples have been collected as arranged with the community leaders.

## 6. Individual Random Selection for Parasitological Examinations and Questionnaires

The study is an augmentation to the current standard of care (annual school-based MDA). With a longitudinal study design, random selection is only required at the individual level for parasitological and epidemiological investigations; all of the individuals in the community will be offered treatment. In each community, following sensitisation, households will be enumerated, with approximate number of residents recorded. Based on the number of households and indicative number of residents in each community, households will then be randomly selected according to a process of selecting every other (odd number houses) household to achieve the sample size. Where a household, or an individual within a household refuses, or is absent during the study, the household will be replaced by the next enumerated household. All the residents within the selected households will be requested to provide urine and stool specimens, and to complete questionnaires. At this stage, individual consent will be elicited, with age, sex, and school-attending status recorded and a unique participant identification number allocated. Selection will proceed until the minimum sample size is achieved. All community members will be invited to receive treatment, with additional consent elicitation by field staff. Treatments will be directly observed, and treatment coverage data will be recorded for all individuals.

For comparison to annual school-based MDA, a junior school (comprising children aged 6–14 years in classes 1 to junior high 3) will be selected at the time the national NTD Control Programme visit occurs. Prior to the deworming, school directors and class teachers will be asked to prepare class lists with the name, age, and sex of the children. Class lists will be imported into Microsoft Excel and each individual will be assigned a computer-generated random number. Individuals will then be selected by study investigators in ascending number order. Individuals who are not present on the day will be replaced by the next individual on the list, until the minimum sample size for parasitological testing is achieved.

## 7. Sample Collections and Questionnaires

Since urogenital and intestinal schistosomiasis, and STH are co-endemic in most endemic communities, selected study participants will be requested to provide both faecal and urine samples. Participants will be given collection pots for urine and stool samples first thing in the morning and asked to return the pots the same day; all the diagnostics will be performed on the same day in order to maintain sample quality. Stool samples will be further stored on ice packs and subsequently in the freezer for any molecular analyses for quality control. Questionnaires will be conducted with these participants at baseline and follow-up by trained field staff; the questionnaires take approximately 30 min to complete, and there are 45 questions in the household questionnaire and 63 questions in the individual questionnaire. Socio-economic and WASH information, previous treatment received, and general morbidity information will be collected through self-reported questionnaire responses within these questionnaires. These data will be used to assess risk factors that are associated with prevalence and/or intensity of schistosomiasis and STH infection, and separately, associations between intensity of infection and morbidity indicators.

## 8. Parasitological Diagnoses

Faecal samples will be examined using the Kato-Katz kit from Sterlitech and compound microscopes (Olympus model CH and Boshida BD-SW30) at ×400 magnification. This will allow for the detection of *S. mansoni* and STH eggs and for the intensity of infection to be calculated as eggs per gram (epg). Urine samples will be visually examined for blood presence (macrohaematuria), tested with Urit 10 V reagent strips for microhaematuria, and filtered to examine the presence and intensity of *S. haematobium* infection. The filtration will involve passing 20 mL of urine through a 25 μm pore Plastok nylon filter housed in a Swinnex filter holder from Fisher Scientific. POC-CCA assays (ICT International) may be used for the detection of *S. mansoni* antigens, and will be conducted according to the manufacturer’s instructions. Samples may additionally undergo qPCR testing at CSIR-WRI or LSTM, using multiplex Taqman DNA assays with species-specific probes (and viral-DNA control) [34]. Quality control checks will be carried out where 10% of the slides will be re-examined by a lead technician to ensure to confirm their positive or negative status. Any discrepancy between the two slide reads will require a re-examination of all the slides up to that point.

## 9. Qualitative Research

Qualitative data will be collected through semi-structured interviews (with those who distribute drugs, including, as appropriate, health workers in the community and school teachers within the schools), and focus group discussions with a purposively sampled range of community members receiving the additional drugs. Semi-structured interviews are conducted with individuals and are structured around key areas of enquiry [35], whereas focus group discussions are held with groups of people with similar characteristics [36]. Community members will include a sample of men, women (including pregnant women) and children who have received the treatment. All qualitative data will be collected by trained and experienced social scientists. The quality of this qualitative data will be assured through training, debriefing, developing rapport with participants, and ensuring the use of open-ended and probing questions [35,37].

Additionally, because deworming treatments are not currently provided to the expanded community groups, qualitative mapping will be conducted to investigate the community structures through which expanded treatments could be provided in the future. Such sites could, for example, include health centres, aid posts, or outreach clinics, and treatments could potentially be included in existing health packages such as antenatal care, child immunisations, vitamin A distributions, maternal health checks, or health education services [13]. Qualitative exploration of community structure to support expanded treatments within the different communities and collaborative design of potential health system interventions, will be conducted following baseline surveying.

## 10. Socio-Economic Evaluation

Economic inquiries will evaluate the potential economic feasibility of the alternative treatment strategies and potential household impact. Inquiries will focus on COUNTDOWN and household expenditures. The societal perspective will be used to identify and measure resource use for both of the intervention components. An economic evaluation study methodology will be used that includes the identification, measurement, valuation of resource use, and calculation of costs (direct cost, indirect costs, and productivity loss). Cost-effectiveness analysis, cost-benefit analysis, and household impact will be used in the economic evaluation of the studies. 

## 11. Eligibility Criteria and Justification

Community members must reside within the identified villages, to reduce the contamination of people joining in the study units whom have not received the intervention. Individuals who do not have a signed consent form, are acutely unwell, school-enrolled children, and people who indicate that they have had deworming treatment within previous 12 months, will be excluded from parasitological analyses. Children aged less than one year will be excluded. For ALB only, women in their first trimester of pregnancy will be excluded, as it is not recommended to be taken. Its safety during second and third trimesters of pregnancy has been agreed [38].

PZQ and ALB treatments will be provided in accordance with WHO guidelines [12,39], following discussion and consent; the team will ensure no one is coerced into taking part. For ALB, standard screening questions, including an estimate of gestation period, are included on the participant register. Where there may be doubt about pregnancy, ALB will not be administered. Preschool-aged children will not be treated without a parent/guardian being present. School-enrolled children will receive annual MDA at school, and therefore do not need to be included for treatment or parasitological analysis.

## 12. Sensitisation and Recruitment

Key programme and academic personnel have been involved in the study and its design since inception. These staff will coordinate the selection of sites and subsequently contact key local persons, including district health and education personnel, school and community leaders. A team leader will be assigned to each field team. Following site selection, team leaders will have primary responsibility for correct sensitization of communities, in accordance with established protocols and etiquettes (for example, contact via chiefs prior to community consultation). Recruitment of participants will be via community consultation, with enrolment of all consenting randomly selected households. Recruitment of school children for parasitological comparison will be via teachers, with take-home consent forms and accompanying information sheets being issued for parental consent. Children randomly selected for parasitological surveillance and questionnaires will be given sample pots for stool and urine samples and asked to bring them back the following day. To recruit participants for the focus group discussions and semi-structured interviews, a sampling frame of all of the eligible participants will be obtained. In line with the theory and practice of qualitative research, a purposive sample will then be taken within this study aiming to ensure maximum variation in age, gender, and socio-economic status, pregnancy, and involvement or otherwise in previous school-based distribution. Qualitative data will be collected until reaching the saturation point (when the research team are confident they have received the full range of experiences and responses) [40].

## 13. Outcomes

The primary outcome is the reduction of prevalence of infection of *S. haematobium* and/or *S. mansoni* from the MDA intervention at study endpoint. For purposes of sample size calculations, a 50% reduction in prevalence has been anticipated (i.e., from 20% to 10% after 12 months). As there are negligible existing studies reporting reductions in prevalence of schistosomiasis following community-based treatment, this number has been based on a drug efficacy study of PZQ syrup administered to preschool-aged children, where the reported cure rate against *S. mansoni* was 50.6% [38].

Secondary outcomes to be measured are:

(i) To quantify the infection of *S. haematobium*, *S. mansoni* and STH in preschool-aged children, out-of-school children, and adults, including pregnant women;
(ii) To assess reductions in intensity of infection of *S. haematobium* and/or *S. mansoni* in study sites at the final timepoint;
(iii) To assess reductions in prevalence and intensity of infection of STH in study sites at the final timepoint;
(iv) To assess treatment coverage at each timepoint;
(v) To investigate associations between the interventions and morbidity, WASH, and demographic factors from the questionnaires;
(vi) To assess access of the interventions in the study sites by participants and critical success factors for future implementation, via qualitative assessment;
(vii) To investigate the cost-effectiveness of the interventions and household equity impact by detailed examination of the resource use and cost changes of delivery within the study settings.

## 14. Data Analysis

The primary outcome for all sites will be assessed in terms of treatment coverage and reductions in infection prevalence. Secondary outcomes, primarily assessment of morbidity, and demographic factors from the questionnaires and qualitative focus interviews, will also be assessed throughout the study and at the end point. Prevalence and intensity of *S. haematobium*, *S. mansoni* and STH infection will be determined by parasitological methods, according to WHO guidelines [39]. In general, the demographic data will be presented as the mean, median, SD and range for continuous data, and number and proportion of subjects in each category for categorical data. For the intensity of infections we will present overall arithmetic means and subsequently arithmetic means, stratified by gender, age-ranges (pre-school children, school-children, adults), and community at each time point. We will also calculate number and proportion of subjects in each class of intensity at each time point. Finally, for the general health data (i.e., morbidity indicators), we will present number and proportion of subjects at each assessment.

More precisely, the analysis of the primary outcome will be as follows: initially descriptive analysis for overall schistosomiasis (urinary or intestinal) prevalence will be presented per time. If the prevalence is more than 5%, descriptive results will also be presented by gender, age-ranges (pre-school children, school-children, adults), and community per time. Adjusted analysis taking into account the nested structure of the data: repeated measurements within study participants within households will be performed through population average logistic regression models that typically use generalized estimating equations (GEE) [41]. Such models will produce an estimate of the log(odds ratio) that compares the average prevalence of infection (averaged across all clusters: individuals within households) at each time point (i.e., time will be included as a covariate) controlling at the same time for individual—(i.e., age, gender and socioeconomic status as derived from socioeconomic variables included in the questionnaires), as well as household-level covariates [42]. Age, gender, and socioeconomic status are also likely confounders, and thus will be tested in the models. In these models, two-way interactions of time with sex, age, and communities will be considered to explore differences in prevalence rates between different groups. Missing data will be handled through multiple imputation or weighted GEE [43,44].

For the secondary outcomes defined as reductions in prevalence of STHs, we will follow a similar approach as the one outlined above for schistosomiasis, and separately for each of the three STH parasites; we will proceed with GEE models if overall prevalence levels were estimated from the descriptive analysis to be above 5% at baseline in each of those. Subsequently, to assess reductions in the different WHO categories of intensities of schistosomiasis and STHs infections, we will employ similar reasoning for different multinomial GEE models for each of these parasites. For the baseline associations between intensities of infection and morbidity indicators, we will consider different multinomial GEE models for each of the examined parasites (i.e., schistosomiasis and STHs). For the baseline associations between prevalence of infection and WASH indicators, we will consider different logistic GEE models for each of the examined parasites (i.e., schistosomiasis and STHs). All analyses will be conducted using SAS version 9.4 for Windows (SAS Institute Inc., Cary, NC, USA). In all of the models mentioned above, the final retained explanatory variables will be based on information criteria between different fitted models (whenever those are available) and Wald tests. Spatial analysis may be employed to create visual prevalence and/or risk maps of schistosomiasis and STH in the study areas (STATA, R, and QGIS/ArcGIS). For schistosomiasis, at the household level, scatter-plots and semi-variograms will be produced to investigate the relationships between household location and proximity of the shoreline. For qualitative analyses, semi-structured interviews and focus group discussions will be analysed using the framework approach [33]. This approach allows for both deductive and inductive theme identification, through two main phases consisting of five steps:

Phase 1: Data management

- Step 1: Familiarisation- the analysis team will read and re-read the verbatim transcripts to ‘familiarise’ themselves with the data and take note of key themes emerging.
- Step 2: Developing a thematic/coding framework through which to sort the data based on original aims and objectives, and any inductive themes identified during familiarization.
- Step 3: Indexing/coding data- the thematic/coding framework will be applied to all the data using NVIVO software, Microsoft Word or Excel.

Phase 2: Data explanation

- Step 4: Charting-data will be lifted from its original context based on its allocation to the coding/thematic framework and placed within a chart.
- Step 5: Mapping variables at household level.

An economic study of NTDs will be conducted under this umbrella protocol and will be published in a separate protocol paper (in submission). The economic study will involve two research questions. The first will focus on the health equity impact of NTDs and MDAs. The focus will be on the impact of schistosomiasis and STH on household socioeconomic variables, such as education and labour market participation. There will also be a focus on the impact of MDAs on health inequalities. The second research question will focus on the cost-effectiveness and value for money of current MDAs and the proposed health intervention. There will be a baseline and a follow-up assessment of the consequences of the interventions. The outcomes of interest include life-years gained, number of symptom days averted, and number of disabilities and deaths averted. The monetary benefits of the proposed intervention will also be estimated. There will be a focus on the impact of the interventions on labour market productivity and outcomes. Costs will be estimated using a combination of both macro- and micro-costing techniques. Direct costs of deworming drugs, logistics and transportation, storage of resources, materials required for preparation and administration of the drugs, screening and diagnosis, number of people tested per diagnostic technique, treatment, materials and equipment for field workers, payment and training of field workers will be estimated. Given that a societal perspective is being adopted, the costs to households will also be estimated. This will include labour market and educational costs. Time spent in receiving diagnosis and treatment will be estimated. Absenteeism from daily activities due to participation in the health intervention will also be estimated.

This series of analyses will enable the simultaneous identification and potential linkage of the epidemiological, parasitological, sociological, gender, and economic factors that are required to determine the effectiveness of scaling-up of MDA against these parasites. These data will be triangulated to assess all implications, contributing to an integrated, holistic approach to health systems strengthening. 

## 15. Sample Size Calculation

Sample size calculations are for the primary outcome, assuming a baseline schistosomiasis prevalence of infection of 20%. Sample size calculations are calculated separately for baseline and follow-up. 

Baseline sample size: This was estimated using a hypergeometric formula (due to small overall community size):

$$n=Nz^{2}\rho q/\left(E^{2}\left(N-1\right)+z^{2}\rho q\right)$$

where: *N* = population size (1500 people); *p* = estimated prevalence (0.2); *q* = 1 − *p*; *E* = accuracy of estimation (0.03); *z* = 1.96 (confidence level 95%).

A 40% buffer was applied to take into account differences in necessary covariates (including age and sex). The baseline sample size per site is 658 people per community of 1500 people. Assuming three communities, 1974 participants will need to be sampled for parasitology and questionnaires.

Follow-up sample size: It is assumed that there will be a post-intervention reduction in prevalence of 50% (i.e., from prevalence of 20% to 10%; see outcomes). This gives an assumed baseline population of 132 ‘infected’, and first-round follow-up of 66 ‘cured’. An additional 20% buffer for age and sex (10% each), and a 10% loss to follow-up between each follow-up have been added. Using a sample size formula for matched proportions:

$$\;n={\left(Z_{\frac{\alpha }{2}}+Z_{\beta }\right)}^{2}\;\times \;\left[p_{0}\left(1-p_{0}\right)+p_{1}\left(1-p_{1}\right)\right]/{\left(p_{0}-p_{1}\right)}^{2}$$

where: *n* = number of individuals required; (*Z*_α/2_ + *Z*_β_)² = test statistic of 7.85, based on standard normal distribution values assuming significance of 0.05 and power of 80%; *p*_0_ = estimated prevalence at baseline (20%); *p*_1_ = estimated prevalence at follow-up (10%).

Assuming a 60% buffer, 314 participants per site will need to be longitudinally followed up each round. Therefore, for three study sites, a total of 942 subjects would be targeted to be followed up.

### 15.1. Informed Consent

Community sensitisation, including the opportunity to ask questions, will occur through the appropriate channels for each community, for example, via village chiefs and after discussion with district health personnel. During community sensitisation, information will be provided about the diseases, their consequences, and treatment, the study, its risks and benefits, and how to participate. Participants will be given the opportunity to ask questions. An information sheet containing this information will be provided to all of the participants or their parent/guardian (if aged less than 18 years). The information sheet will be provided in English, and read out by local translators in the case of illiteracy since there are several languages spoken in the area. The information sheet contains contact details for research staff in case participants have subsequent questions. Information will be repeated and clarified before consent is recorded. Participants will then be enrolled in the study and asked to sign or fingerprint a consent form that will also be read out in the case of illiteracy. The consent forms separately cover consent for parasitological specimens, administration of questionnaires, and deworming treatments in case participants consent for some, but not all, procedures. Participants will not be coerced, rushed, or forced to take part at any time. Children under 18 years will have parental/guardian consent. Children whose parents/guardians have provided consent may also indicate their assent to participate. If a parent/guardian has not provided consent, children will not be able to provide their own assent. In such cases where a parent/guardian has provided consent but obvious dissent is expressed by the child, the child will not be forced to participate. All of the individuals will be free to opt out of the studies at any time, with no censure. No participant will be asked to provide parasitological or epidemiological information, or participate in quantitative and qualitative methods, without a signed consent form. No participant will be eligible to receive treatment without signed consent.

### 15.2. Quality Assurance

For parasitological diagnoses, slides will be examined in duplicate with 10% quality control assessments by microscopy team supervisor. Questionnaires have been developed based on standardised questions that are administered elsewhere. Field staff will receive training in questionnaire administration. Subsequent data quality checks will be periodically undertaken by an epidemiologist, with additional field training provided as required. Questionnaires will additionally be pre-tested with field staff, and piloted on the first ten participants to investigate the field-utility for the interviewer and reliability of the questions. Any adjustments will be noted and discussed with an epidemiologist before changes are made. Pilot results will only be included in analyses if questions are not amended. Data management and cleaning of participant registers and all parasitological and questionnaire data will be overseen by an epidemiologist. The microscopy team supervisor and the epidemiologist are independent to eliminate bias in reading results.

For qualitative research methods, extensive training will be provided to the research assistants in the aims and objectives of the study, as well as conducting interviews and focus group discussions. Discussion guides will be piloted with participants outside the study villages to ensure that the meaning of the questions is clear and open and probing questions are used. If permission is provided, all focus group discussions and interviews will be taped. They will then be transcribed and translated into English. A proportion of these transcriptions will be checked to ensure the quality of the translation and transcripts will be shared with the broader team for critical review. A research diary will be kept providing a full record of the process of data collection and analysis. To ensure validity of the results, deviant case analysis will be undertaken and the ongoing analysis will reflect these results. The researcher will triangulate the results from the focus groups discussions and the semi-structured interviews, looking for patterns of convergence to develop or corroborate the overall interpretation [34].

A participant register will be used at each community site, and school enrolment lists at each school site; a unique person identifier will be allocated from these registers. The unique identifier will be used on parasitological samples and questionnaires to maximise participant confidentiality. All data will remain anonymised; paper-based data will be securely stored in locked cabinets in country COUNTDOWN offices, and electronic versions will be secured with encryption software. Of particular note, all PZQ and ALB distribution is in accordance with WHO guidelines and involves no increased risk to patients by being delivered in this setting. Upon study exit, all of the participants will be offered a final round of MDA.

## 16. Discussion

The ultimate aim of this research is to document the disease burden in currently overlooked demographical groups and to demonstrate the feasibility of enhancing an existing intervention package and create conditions for better treatment coverage of schistosomiasis and STH control strategies. This implementation research will be of importance in demonstrating schistosomiasis prevalence in preschool, out-of-school children, and adults, including pregnant women in regions of Ghana, and ensuring more equitable access to future interventions. Using a broad range of complementary, multidisciplinary skillsets, the study will be of primary importance in assessing the disease reductions, feasibility, and acceptability of alternative treatment strategies on schistosomiasis and STH. This is likely to have considerable research and political value to contribute evidence for national programme policy development within the country, and, more broadly, WHO policy development. This will contribute to the evidence base for the development of post-2020 implementation priorities. The political value of including STH is also important, as results from this COUNTDOWN study will be available in a similar timeframe to other trial results, thereby augmenting the evidence base with data from additional countries and contributing to a multi-stakeholder push for expanded treatments to at-risk individuals. Removing drug access bottlenecks will facilitate a larger proportion of infected, or at-risk, individuals being able to access treatment and promote universal health coverage.

## Supplementary and Materials

The data from the COU**NTD**OWN studies will be made freely and publicly available at the completion of the studies and analysis of study outcomes. This is anticipated at the end of 2019, subject to approved data sharing agreements.

## Abbreviations

ALB	Albendazole
CSIR-WRI	Council for Scientific & Industrial Research-Water Research Institute, Accra, Ghana
GHS	Ghana Health Service
HIV	Human immunodeficiency virus
LSTM	Liverpool School of Tropical Medicine
MDA	Mass drug administration
NTD	Neglected tropical disease
POC-CCA	Point-of-care circulating cathodic antigen tests
PZQ	Praziquantel
qPCR	Quantitative polymerase chain reaction
SCORE	Schistosomiasis Consortium for Operational Research and Evaluation
STH	Soil-transmitted helminth
WASH	Water, sanitation and hygiene
WHO	World Health Organization
